# Elevated Background Noise in Adult Attention Deficit Hyperactivity Disorder Is Associated with Inattention

**DOI:** 10.1371/journal.pone.0118271

**Published:** 2015-02-18

**Authors:** Emanuel Bubl, Michael Dörr, Andreas Riedel, Dieter Ebert, Alexandra Philipsen, Michael Bach, Ludger Tebartz van Elst

**Affiliations:** 1 Department of Psychiatry and Psychotherapy, Albert-Ludwigs-University of Freiburg, Hauptstrasse 5, Freiburg, Germany; 2 University Eye Hospital, Albert-Ludwigs-University of Freiburg, Killianstrasse 5, Freiburg, Germany; Central Institute of Mental Health, GERMANY

## Abstract

**Background:**

Inattention and distractibility are core symptoms of attention deficit hyperactivity disorder (ADHD). Still the neuronal organization is largely unknown. Previously we studied the electrophysiological activity of a distinct neuronal network—the retina—and found no change in stimulus-driven neural activity in patients with ADHD. However there is growing evidence for an elevated non stimulus-driven neural activity, or neuronal background noise, as underlying pathophysiological correlate. To further examine the biological bases that might underlie ADHD and problems with inattention, we performed a new analysis to test the hypothesis of an elevated background noise as underlying neuronal correlate for ADHD and problems with inattention in humans. A direct measure of background noise in patients with ADHD has not been described yet.

**Methods:**

The retinal background noise was assessed based on pattern electroretinogram (PERG) data in 20 unmedicated ADHD patients and 20 healthy controls. The PERG is an electrophysiological measure for retinal ganglion cell function. ADHD severity was assessed by interview and questionnaire.

**Results:**

Noise amplitude was significantly higher (138%) in patients with ADHD compared to the control group (p = 0.0047). Noise amplitude correlated significantly with psychometric measures for ADHD (CAARS) especially inattention (r = 0.44, p = 0.004).

**Conclusions:**

The data provide evidence that an elevated background noise is associated with symptoms of inattention in ADHD and support the use of therapeutic interventions that reduce noise and distraction in patients with ADHD.

## Introduction

Attention deficit hyperactivity disorder (ADHD) is one of the most common mental disorders in children and adolescents (8–12%) with a high persistence into adulthood (4–10%)[1,2]. Patients with ADHD are frequently characterized as distracted and inattentive. However, the underlying neuronal correlate is poorly understood. An elevated noise or “non stimulus-driven neural activity” and its modulation by dopamine has been proposed as underlying pathophysiological mechanism and treatment target in ADHD, particularly in the prefrontal cortex (PFC) [3–7]. Medication like methylphenidate elevates dopamine, effectively reduces distractibility, and improves attention in patients with ADHD [8–11]. Animal studies suggest that a basic mechanism of action of dopamine is the reduction of non stimulus-driven neural activity or noise [12–14].

From a functional viewpoint, a reduction in noise is proposed to lead to a decrease of irrelevant information processing and a reduction in distractibility [4,9,15]. However, to the best of our knowledge, no study has directly measured noise in patients with ADHD.

One approach to objectively assess noise in humans and animals is the assessment of the pattern electroretinogram (PERG) evoked by phase-reversing checkerboard stimuli. It offers an easy, direct access to the neuronal network of the retina, which receives minimal influence from higher cortical regions and provides information on early signal processing. The PERG is an objective electrophysiological response, recorded at the cornea in response to visual pattern stimulation [16,17]. It mainly represents the activity of the retinal ganglion cells [18,19] and thus can serve as a surrogate marker of neuronal information processing in the retinal network from the photoreceptors to the optic nerve.

The visual system is a prominent candidate in exploring neurobiological processes underlying attention [20–22]. For example, in their landmark work, Wurtz and Goldberg (1972) revealed a direct neural correlate of attention, highlighting the specific relevance of the superior colliculus and the visual system in the shift of attention [23]. The superior colliculus has also been targeted in the exploration of the mechanism of action of stimulant medication such as methylphenidate and d-amphetamine on neuronal activity in rats, with a strong reduction of noise after treatment, without a drug effect on the signal amplitude [24]. This is consistent with previous work on other brain regions like the PFC [7,25]. Recently, we reported unaltered PERG amplitudes to contrast stimuli in unmedicated patients with ADHD compared to controls [26], this contrasted with reduced signal amplitude in depressed patients [27]. However, it remains open if background noise or non stimulus-driven neural activity is altered in patients with ADHD rather than signal-related processing. This could result in an increase of irrelevant information processing and symptoms of distractibility in patients with ADHD. To evaluate this hypothesis, we performed an additional analysis to explore whether an elevated retinal noise might be evident in patients with ADHD. As surrogate measure for noise or “non stimulus-driven neural activity” we used spectral magnitude close to, but not confounded by the dominant response frequency (which relates to the stimulation frequency) of the PERG. For brevity, we will use the term “background noise” for this measure throughout this text.

## Materials and Methods

### Ethics Statement

The research protocol was approved by the ethics board of the Albert-Ludwigs-Universität Freiburg. Written informed consent was obtained from all participants after the study was fully explained to them.

### Subjects

The PERG recordings were conducted at the Eye Center of the University Medical Center of Freiburg. Twenty unmedicated patients with ADHD (10 male and 10 female; 33.5±12.5 years of age) were identified at the ADHD program of the Department of Psychiatry and Psychotherapy of the University Hospital of Freiburg (Table 1). The population is identical to the group of patients reported earlier with respect to PERG-based contrast gain [28].

**Table 1:** Group comparison for demographic characteristics.

	Controls(n = 20, f = 10)	ADHD(n = 20, f = 10)
	Mean±SEM	Mean±SEM
Age	33.5(2.7)	33.8(2.8)
Smokers	2	4
Mean years of schooling	12.9(0.15)	12.6(0.2)

Abbreviations: n = number total, f = number of female, SED = standard error mean.

The Freiburg University Center is one of only a few national centers for the diagnosis of developmental disorders in adulthood (ADHD, autism, and tic disorders)[29]. The diagnostic procedures for ADHD and autism follow NICE guidelines, which include a careful developmental history; a history by parents or caregivers is the centerpiece of establishing the clinical diagnosis by very senior and experienced consultant psychiatrists. In addition, records from early childhood were routinely integrated into the assessment, especially interviews with parents and regular reports of the students’ progress.

Participants met the DSM-IV criteria for ADHD, classified as either ADHD of the combined or predominantly inattentive type. We excluded all other first axis, major psychiatric disorders, developmental disorders like autism spectrum disorder (ASD) or other neurological, ophthalmological, or medical conditions, except for correctable refractive errors. Senior consultant psychiatrists (board certified, integrated into the resident and students training program on diagnosing psychiatric (disorders according to ICD-10 and DSM) assessed the ADHD diagnosis on the basis of a detailed semi-structured as well as structured psychiatric interview (SCID, ADHD Diagnostic Checklist) and specifically detailed developmental history following recommendation of respective NICE guidelines for the diagnosis of ADHD and ASD in adulthood[30–33].

Furthermore, the ADHD Diagnostic Checklist corresponding to the DSM-IV-criteria for ADHD adapted for the special needs for adults, as proposed by the German Medical Association (ADHD Diagnostic Checklist), was also carried out[31,32,34]. This diagnostic procedure also integrated common psychiatric and somatic differential diagnoses as well as the patients’ medical histories. Consultants were specially trained to differentiate between ADHD and ASD, since they were integrated in the ADHD and ASD outpatient program. All patients were also psychometrically assessed using the Wender Utah Rating Scale and Conners’ Adult ADHD Rating Scales-Self-Report: Long version(CAARS-S:L) and the Becks Depression Inventory (BDI)(Table 2) [35–37]. Eleven patients fulfilled the criteria for ADHD of the combined type (DSM-IV: 314.01) and nine for the predominantly inattentive type (DSM-IV: 314.00). The mean BDI in patients was 11.7± 2.1 SEM and 2.5± 0.4 SEM in the control group. When some patients presented with higher BDI scores we ensured in the diagnostic assessment that no patient suffered from a depressive episode. Both groups did not differ in mean duration of schooling (patients: 12.6± 0.2 SEM; controls: 12.9± 0.15 SEM). The control group consisted of 20 age- and gender-matched healthy subjects (10 males and 10 females; 33.8±12.1 years of age) without a history of neurological or mental disorders, all of whom scored in the normal range on the ADHD Diagnostic Checklist and CAARS (Table 2). All subjects had a visual acuity of above 20/25 with appropriate correction at the distance used for visual stimulation [38].

**Table 2:** Group comparison for psychometric characteristics and Correlation with noise.

	Controls(n = 20, f = 10)	ADHD(n = 20, f = 10)	Correlation with noise	
	Mean±SEM	Mean±SEM	r	p
CAARS				
A. Inattention**	6.3(0.8)	21.2(1.4)	0.44	0.005
B. Hyperactivity*	4.5(0.6)	19.5(1.3)	0.35	0.026
C. Impulsivity	5.5(0.6)	17.7(1.8)	0.25	0.115
D. Self-concept	3.5(0.6)	9.8(0.8)	0.17	0.268
E. DSM-IV inattentive**	2.5(0.4)	15.5(1.1)	0.44	0.004
F. DSM-IV hyperactivity	3.1(0.6)	12.1(1.6)	0.28	0.076
G. ADHD symptoms total*	5.6(0.8)	27.7(2.5)	0.38	0.015
H. ADHD index*	5.8(0.7)	19.6(1.5)	0.33	0.041
ADHD Diagnostic Checklist				
Intattention **	1.9(0.3)	7.5(0.3)	0.44	0.005
Hyperactivity	1.6(0.5)	6.6(0.6)	0.28	0.085
WURS		48.5(3.5)		
BDI	2.5(0.4)	11.65(2.1)	0.11	0.49

Abbreviations: n = number total, f = number of female, SEM = standard error mean; CAARS: Conners’ Adult ADHD Rating Scale; DSM: Diagnostic and Statistical Manual of Mental Disorders. WURS: Wender Utah Rating Scale; BDI: Becks Depression Index.

*p< 0.05;

**p< 0.01

### Stimulation and recording

For stimulation, recording, and analysis, we used the EP2000 system [39]. In a dimly lit room, the stimuli were generated with a resolution of 800 × 600 pixels at a frame rate of 75 Hz and displayed on a monitor covering a field size of 32° × 27.0° at an observation distance of 57 cm with a mean stimulus luminance of 45 cd/m^2^. Our patients were refracted as necessary for the observation distance. To ensure appropriate fixation and accommodation, they reported digits that appeared in random intervals in place of the fixation target.

To evoke the PERG, a sequence of five checkerboard stimuli with a check size of 0.8°, contrast-reversing at 12.5 reversals per second, was presented with Michelson contrasts of 3.2%, 7.3%, 16.2%, 36%, and 80%. Each contrast level was presented for 10 s, and then the next contrast was applied, finally returning to the first contrast level. This interleaved sequence was presented until 80 artefact-free trials per contrast (0,96 length each, containing 12 responses) were accumulated. The interleaved blocking ensured that any sequential effects (e.g., fatigue) were distributed equally across all contrast values. This protocol was repeated once, and further analysis was based on the vector average of each pair of recordings.

The PERG response was recorded simultaneously from both eyes using DTL electrodes [40] placed at the lower limbus of each eye. These were referenced to gold cup electrodes at the ipsilateral outer canthi; one earlobe was grounded. Subjects were instructed to blink infrequently during recording and to maintain a relaxed pose. Sweeps exceeding ±130 μV were rejected as artefacts, and the number of artefacts per condition was saved with the PERG data.

The signals were amplified, filtered (first order 0.5–100 Hz), and digitized at 1 kHz with a 16-bit resolution. To prevent temporal aliasing, all timing (stimulation, analog sampling, and sweep length) was related to the stimulus monitor frame rate [41,42]. The recording’s total duration was approximately one hour per subject.

### Data analysis

Offline, all traces were first detrended (trend mainly remaining from blink excursions) by calculating a linear regression along the trace and subtracting it (to avoid sawtooth artifacts mimicking background noise) [41,42]. Then the magnitude spectrum was calculated by discrete Fourier transform. Based on the analysis interval of 0.96 s, the spectrum starts at 1.04 Hz, is spaced in 1.04 Hz intervals, and specifically contains responses at the reversal rate (12.5 Hz), its harmonics (25 Hz, 37.5 Hz, etc.) and background noise (non stimulus-driven neural activity) at all frequencies, and occasionally mains interference at 50 Hz. From this spectrum, we estimated the background noise as the average of the two spectral magnitudes next to the target signal at 12.5 Hz (at 11.46 and 13.54 Hz). The non-linear superposition of noise and response magnitude was first analyzed by Strasburger [43] in the context of steady-state VEP recording, which also applies to steady-state PERG recording. When strict integer relations of all pertinent frequencies are selected (see note on temporal aliasing above), this spectrum contains response power only at the stimulus frequency and its harmonics, consequently as noise estimate Norcia et. al. [44] used one spectral line, offset by 2 Hz from their response frequency. The average of two adjacent frequencies above and below the response frequency is an even better estimator of background noise [45], so this was employed here. The overall background noise ratio was established by the average noise level across all contrast levels and both eyes. Possible differences in retinal noise between the two groups were calculated using Wilcoxon rank sum test. Further, Pearsons’s rank correlation was used to assess the relationship between neuronal noise and psychometric properties. A p-value of 0.05 was chosen as the criterion of significance.

## Results

### Background noise group comparison

Table 1 illustrates that both study groups were well matched with respect to age, gender and mean years of schooling. As expected, there were significant differences in psychometric scoring between the two groups (Table 2).

**Fig 1:**
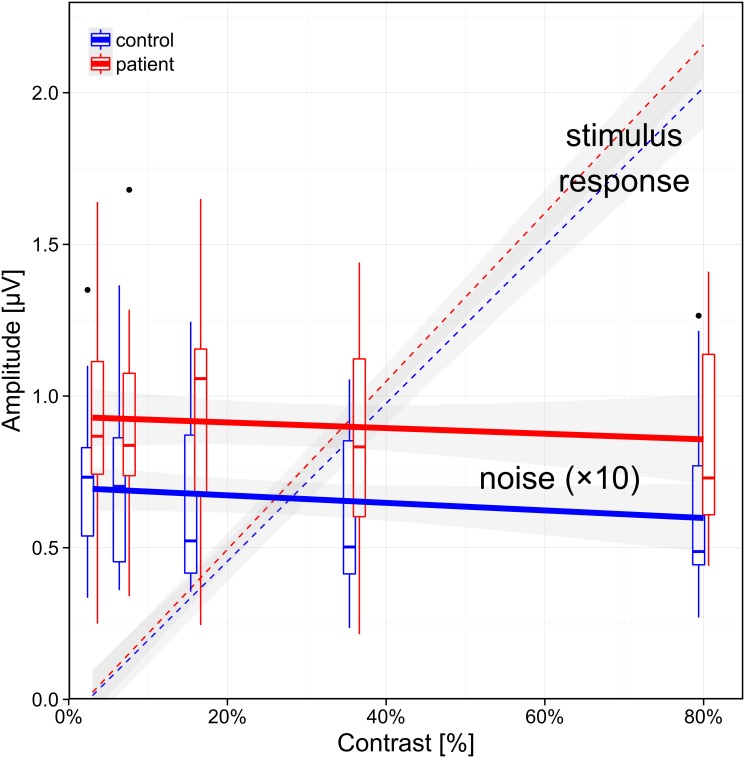
Relationship of PERG background noise to stimulus contrast. Controls are displayed in blue, patients with ADHD in red. The thick, nearly horizontal lines represent a first-order model fit to the pertinent data points, the gray-shaded areas indicate its ±SEM interval. The distribution of the original data points can be gleaned from the box plots, where the median is indicated by short thick horizontal lines, the box covers the 25–75% percentile range, the whiskers indicate the range, outliers are indicated with dots. The background noise is markedly higher (138%) in patients with ADHD (red) compared to the controls (blue, p<0.005) and does not depend on stimulus contrast (lines are almost horizontal). This differs from the PERG stimulus response, which we added for comparison as dashed lines (data from [26]); the PERG response amplitude rises linearly with stimulus contrast.

Fig. 1 shows the group difference of background noise for the two groups. The two lines are nearly horizontal indicating that background noise does not depend on stimulus contrast.

**Fig 2:**
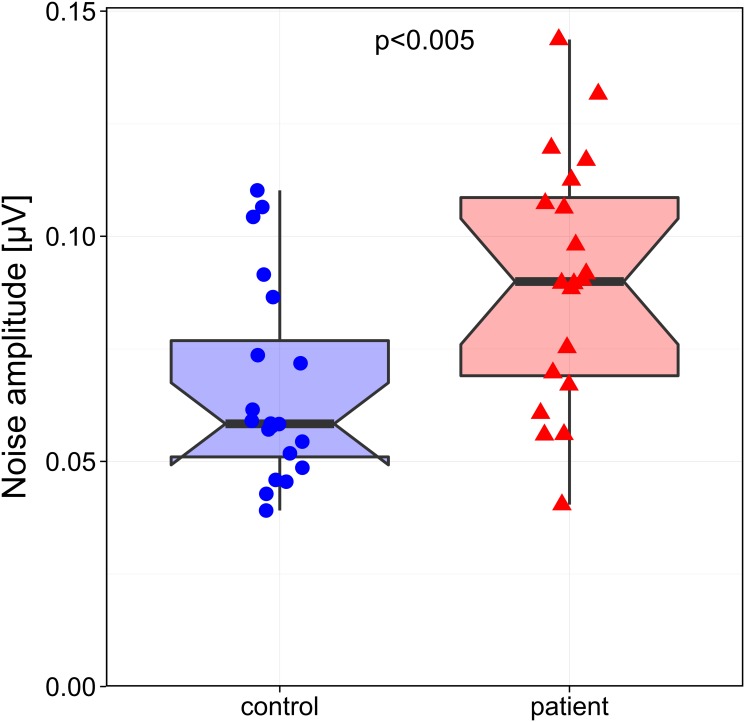
Group comparison of mean neuronal noise. Boxplot showing the comparison of background noise in μV (y axis) between healthy control subjects (HC) and patients with ADHD; noise was averaged across all contrast levels per subject. Patients presented with a significant elevated background noise (p<0.005). Left: control group; right: patients with ADHD. Small disks or triangles, respectively, represent the individual data points. Boxplot details as in Fig. 1, the notches represent a 95% confidence interval for the medians.

In patients with ADHD background noise is markedly (138%) and significantly higher than in the control group (p = 0.0047) [control group: mean background noise 0.066±0.005 µV (SEM); patients: mean background noise 0.091±0.006 μV, see Table 3 and Fig. 2].

**Table 3:** Amplitude of neuronal noise and number of artefacts.

	Controls n = 20	ADHD n = 20	
	Mean±SEM	Mean±SEM	P-value
Neuronal noise	0.066±0.005 μV	0.091±0.006 μV	0.0047
Number of artefacts	115.9±20.6	102.1±17. 7	0.62

While neuronal noise differs significantly between the groups, there was no significant difference in artifacts, thereby indicating that restlessness, frequent eye movement, or cooperation of subjects did not confound the results.

### Correlation of background noise with ADHD Symptoms

To test whether background noise was associated with the severity of ADHD symptoms in subjects, background noise was correlated with the severity of ADHD symptoms obtained with the CAARS.

The CAARS scales for inattention (r = 0.44; p = 0.005), hyperactivity (r = 0.35; p = 0.026), DSM-IV inattentive symptoms scale (r = 0.44; p = 0.004), DSM-IV ADHD symptoms total (r = 0.38; p = 0.015), and ADHD index (r = 0.33; p = 0.041) correlated significantly with background noise; however, there was no significant correlation with symptoms of impulsivity (r = 0.25; p = 0.115), problems with self-concept (r = 0.17; p = 0.268), and DSM-IV hyperactive-impulsive symptoms (r = 0.28; p = 0.076).

In the DSM-IV Checklist, we again found a correlation with the inattentive items (r = 0.44, p = 0.005), but no significant correlation with the hyperactive items (r = 0.28; p = 0.085).

Separate group comparison (patients, healthy controls) showed no significant correlation in the CAARS or ADHD Diagnostic Checklist.

### Correlation of background noise with Becks Depression Inventory (BDI) and age

In previous studies we reported an association between severity of depression and retinal contrast gain. Neither the BDI nor subjects age was correlated with background noise.

### Artefacts

Artefacts are a measure of recording quality and can estimate whether the results might be biased due to aspects such as restlessness, frequent eye movement, or cooperation of subjects. We found that the number of artefacts did not differ significantly between the two groups. In fact, a slightly greater number of artefacts were found among the control group, with a mean of 115.9 compared to 102.1 in the patients group (t = 0.504, p = 0.62).

## Discussion

This study provides evidence for an elevated retinal noise in ADHD. Background noise derived from PERG was higher in ADHD patients than in the control group and correlated with measures of inattention. The retina is a distinct neuronal network without immediate top-down regulation from higher cortical regions. Here, an elevated background noise suggests a compromised neuronal information processing and could explain why patients with ADHD are easily distracted.

Our results support the hypothesis of an increased background noise as the underlying pathophysiological mechanism in ADHD [4,46]. Consistent with this hypothesis are findings from positron emission tomography (PET) research, where methylphenidate reduces task related glucose utilization in healthy adults as a correlate for cerebral efficacy or background noise reduction [15]. Methylphenidate acts on the dopaminergic system by increasing dopamine levels [9]. In patients with ADHD, a reduced dopamine function is elicited, which could explain one mechanism of action of methylphenidate by elevating dopamine and reducing background noise [47,48]. As reported here, an elevated background noise could indicate a reduced dopamine function in patients with ADHD [49]. Indeed, background noise is modulated by the dopaminergic system in other brain circuits [4]. Studies from different brain regions in animals, like the PFC or the striatum, reveal that dopamine reduces the activity of spontaneously active neurons before affecting the stimulus amplitude, thereby reducing background noise [7,12,50]. This has been postulated to be mediated by the stimulation of dopamine receptor D_1_(DRD1) [3,4]. In line with this, moderate DRD1 stimulation in monkeys was associated with noise reduction in the oculomotor delayed response task without changing the signal amplitude. At the same time overstimulation reduced both background noise and response amplitude, thereby implying that our finding may reflect an alteration in the DRD1 pathway [7,51,52].

The positive correlation of background noise with the ADHD index and the DSM-IV ADHD symptoms total score also supports the hypothesis that an increased background noise is consistent with ADHD. The ADHD index discriminates adults who are likely to be diagnosed with ADHD, while the DSM-IV ADHD symptoms total summarizes the number of DSM-IV symptoms of a subject. Some authors suggest that these two scales distinguish individuals with clinical levels of ADHD symptoms, irrespective of whether they have been diagnosed before [53]. The association between the probability to be diagnosed with ADHD and background noise highlights the possible role this signal might play in the pathophysiology of ADHD.

**Fig 3:**
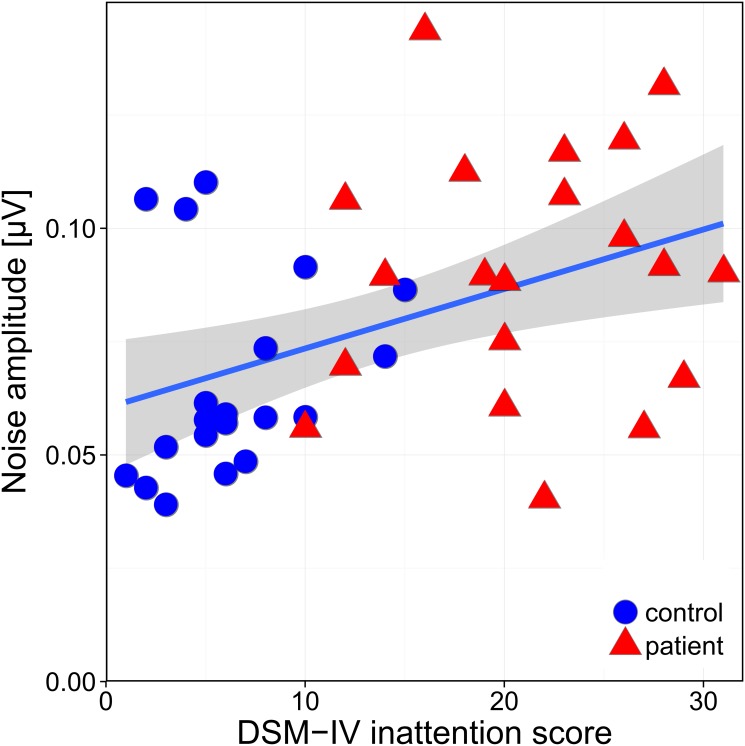
Correlation between background noise and DSM-IV inattentive symptoms, measured with the CAARS-S-L. Healthy controls are represented by blue disks, patients with ADHD by red triangles. Higher ADHD symptoms of inattention are associated with higher background noise (r = 0.44; p = 0.004). The correlation is represented by the line, while the gray-shaded area indicates its ±SEM interval.

Furthermore, in our study, background noise significantly correlated even stronger with symptoms of inattention (CAARS: DSM-IV inattentive symptoms and inattention problems) (Fig. 3), thereby providing evidence that an elevated background noise is linked to inattention. These results are in line with the clinical finding that patients with ADHD are easily distracted, particularly in situations without salience or strong stimuli. A previous imaging study revealed that methylphenidate enhanced the salience of mathematical tasks by enhancing dopamine [54]. Consistent with this are findings from different PET studies, where higher dopamine levels in the ventral striatum were associated with lower inattention scores both in adolescent and adults with ADHD [55,56].

Interestingly, retinal signal processing is known to be modulated by dopamine (DRD1 and DRD2 pathway)[51,57]. For example, it is involved in lateral inhibition or contrast detection [58,59]. The association between retinal noise to contrast stimuli and inattention at the retinal level could indicate an alteration in the retinal dopaminergic system or dopamine system in general. Dopamine has been found to modulate background noise in different brain regions in animals [12,60]. An elevated background noise as early in the processing stream as in the retina provides further evidence that an elevated background noise might not be limited to only one brain region in ADHD.

### Clinical implications

Our finding of an elevated retina noise and the association with inattention may have clinical relevance. While the retina is a very early stage in sensory signal processing, bottleneck alterations there could already have important consequences for attention and distractibility. The involvement of the retina in ADHD supports the use of interventions that reduce distractibility or enhance the salience of tasks [61]. Interventions such as expanding attention span, distractibility delay, and modifying the environment were found to improve performance in patients with ADHD [62]. Pharmacological treatment, for example with methylphenidate, reduced distractibility and inattention and has an important impact on the visual system and distractibility simultaneously, at least in animal models of the disease [24,63–65].

### Limitations

Patients with ADHD are hyperactive and the elevated background noise could be a result of motor activity. However, this is unlikely here since elevated motor activity would result in an increase of artefacts. The artefacts did not differ between the two groups; on the contrary, the number was even lower in the ADHD group. Some additional limitations have to be addressed. Is it possible that patients with ADHD could not perform the task due to problems with their attention? This is not likely since the confounding effect of attention was minimized using an electrophysiological measure that is largely independent of attentional processes. Subjects basically had to watch a computer screen with checkerboard patterns of different contrasts. We ensured that participants cooperated, fixated, and focused by asking them to read out randomly presented digits from the screen center which was performed well by both patient and the control groups. We did not find a correlation of our measures of retinal noise with DSM-IV hyperactivity, self-concept or impulsivity scores in the CAARS, which could indicate either that the CAARS scores were low so the sensitivity for a correlation was not given or the pathophysiology underlying hyperactivity and impulsivity is not represented by physiological alterations at the retinal level, respectively is of different origin.

There was no significant correlation between CAARS scores and background noise in the separate group analysis (ADHD or controls only). Fig. 3 illustrates that this is probably due to a lack of statistical power, suggesting that the sample size of each group was to small for the separate group analysis to become significant. Alternatively the background noise does not correlate with measures in the CAARS. However, we think this is unlikely since there is an overlap and continuum between the two groups, both in background noise as well as in CAARS scores indicating an association between both.

### Conclusions

This study shows an elevated background noise in patients with ADHD compared to controls. Our findings and that of other studies suggest that an elevated background noise might be an important pathophysiological mechanism underlying problems with inattention and distraction in ADHD which can be accessed at the level of the retina.
